# Longitudinal Assessment of Changes in Lifestyle Behaviors and Body Weight from Precollege to Adulthood

**DOI:** 10.3390/nu18030389

**Published:** 2026-01-24

**Authors:** Sujata Dixit-Joshi, Christina D. Economos, Peter J. Bakun, Caitlin P. Bailey, Jeanne P. Goldberg, Erin Hennessy, Nicola M. McKeown, Susan B. Roberts, Gail T. Rogers, Daniel P. Hatfield

**Affiliations:** 1 Gerald J. and Dorothy R. Friedman School of Nutrition Science and Policy, Tufts University, Boston, MA 02111, USA; sujata.dixit_joshi@tufts.edu (S.D.-J.); christina.economos@tufts.edu (C.D.E.); peter.bakun@tufts.edu (P.J.B.); jeanne.goldberg@tufts.edu (J.P.G.); erin.hennessy@tufts.edu (E.H.); susan.roberts@tufts.edu (S.B.R.); gail.rogers@tufts.edu (G.T.R.); 2 Milken Institute School of Public Health, George Washington University, Washington, DC 20052, USA; cbailey@wellesley.edu; 3 Sargent College of Health and Rehabilitation Sciences, Boston University, Boston, MA 02215, USA; nmckeown@bu.edu; 4 Geisel School of Medicine, Dartmouth College, Hanover, NH 03755, USA; 5 FHI 360, Washington, DC 20037, USA

**Keywords:** lifestyle behavior, body weight changes, precollege, adulthood, longitudinal analyses

## Abstract

**Background/Objective**: Lifestyle behaviors evolve with age and are driven by biological requirements (e.g., growth and development) and environmental changes (e.g., living and working situations), and they interact bidirectionally with health. Few studies have tracked these behaviors from emerging adulthood into later adulthood. This study examines changes in lifestyle behavior patterns from precollege to adulthood and their association with weight trajectories. **Methods**: Between 1998 and 2007, 4783 incoming undergraduate students at a northeastern US university completed a health survey. In 2018, 970 completed a follow-up alumni survey. Latent class analysis (LCA) was used to categorize respondents into five lifestyle patterns: stable healthy, stable moderately healthy, stable minimally healthy, worsened, or improved. BMI trajectories were similarly classified into five weight status patterns. Associations between LCA lifestyle patterns and weight were examined using ANCOVA. **Results**: The most common lifestyle pattern was stable moderately healthy (36.7%). Over 11–20 years, 31.7% of respondents experienced a decline in lifestyle behaviors, and 18.6% improved. During this period, the prevalence of overweight more than doubled (12% to 26%), and obesity quadrupled (2% to 8%). Transitioning to a higher BMI category was noted in 34.9% of those with a stable minimally healthy lifestyle compared with 15.9% among those with a stable healthy lifestyle. **Conclusions**: Early lifestyle behaviors have long-term implications for weight status. Initiatives that promote the adoption and maintenance of healthy behaviors from precollege through adulthood might reduce obesity risk.

## 1. Introduction

The prevalence of overweight and obesity among adults in the United States has increased over time, and currently, 31.1% of US adults are overweight, and 42.5% of adults have obesity, including 9% with severe obesity [1,2]. Further, it is estimated that by 2035, 58% of adults will have obesity, underscoring the need to halt and reverse these weight status trends and avert resulting health risks such as cardiovascular disease, type 2 diabetes, and metabolic syndrome [3] and associated health care costs [4]. Recent estimates indicate that the annual cost to treat diet-related chronic diseases is $1.1 trillion [4], which is equivalent to the total amount Americans spend on food each year [5].

Critical periods for the development of obesity include gestation, early infancy, early childhood, early adolescence, and emerging adulthood [6], with the most significant weight gain occurring during emerging adulthood, defined as ages 18 to 25 years [7,8]. Emerging adults navigate increased independence, identity exploration, and shifting social roles [9], which may be accompanied by changes in diet, physical activity, and sleep patterns. These shifts in lifestyle behaviors may play a critical role in weight status and cardiometabolic health.

In 2023, about 63% of recent high school graduates were enrolled in college or university [10]. This transition introduces new lifestyle challenges, including moving away from home, increased personal responsibilities, and changes in social support systems [9]. These changes frequently lead to the adoption of suboptimal health behaviors, including poor dietary choices, decreased physical activity, and insufficient sleep [11,12,13]. Collectively, these lifestyle behaviors can contribute to weight and adipose tissue gain and reductions in lean mass, leading to unfavorable alterations in body composition that diverge from healthy weight trajectories [14,15,16,17].

Persistence of unhealthy lifestyle behaviors beyond college years correlates with increased cardiometabolic risks, including obesity, hypertension, and insulin resistance [18]. While there is acknowledgement that lifestyle behaviors adopted during emerging adulthood often persist in later years, limited studies have examined collective lifestyle patterns and their long-term impacts on weight status. This study examines the longitudinal association between changes in lifestyle behavior patterns from precollege to adulthood and weight trajectories over a 11-to-20-year period.

## 2. Materials and Methods

Surveys: This study utilized a precollege survey administered to incoming undergraduates at a mid-sized university in the northeast of the US from 1998 to 2007, followed by a 2018 alumni survey conducted 11 to 20 years later. Precollege surveys were distributed in paper format via mail and returned using prepaid envelopes. Alumni surveys were administered electronically to respondents with current email addresses available through the university’s office of alumni affairs (*n* = 4127).

Measures: Both the precollege and alumni surveys assessed demographic variables and lifestyle behaviors, including diet, physical activity, sleep, and height and weight.

Dietary measures, including daily intake of fruits, vegetables, and dairy, were measured using investigator-developed items and captured consumption in the past 24 h. Response options ranged from 0 to more than five servings; these were collapsed into three categories for analyses: low (0–1 serving), moderate (2–3 servings), and high (4+ servings). Self-reported dietary patterns were characterized as omnivorous, pescatarian, lacto-ovo vegetarian, and vegan and dichotomized into unrestricted (omnivorous) and restricted (pescatarian, lacto-ovo vegetarian, and vegan), based on reported associations with more favorable health outcomes [19,20]. This dichotomous variable does not imply that restricted diets are always healthier than non-restrictive ones; instead, it is intended to serve as a contextual indicator of dietary patterns. Similarly, while dairy intake is associated with higher intakes of several key nutrients, higher dairy intake may not always correlate with higher dietary quality; therefore, we did not treat it as a standalone marker of dietary healthfulness but rather as one of several food group indicators commonly used in population nutrition surveillance to reflect dietary adequacy and patterning.

Physical activity data were gathered differently at two timepoints. The precollege survey used an investigator-developed item with open-ended responses to capture activities completed, frequency (times per week), and duration (minutes/session). The alumni survey used the International Physical Activity Questionnaire (IPAQ) [21], which quantifies and categorizes activity by intensity level. At both points, total weekly PA minutes (including light activity) were calculated and classified into three categories: low (<200 min), moderate (200–400 min), and high (>400 min), based on the tertiles of the precollege distribution. Sensitivity analyses exploring alternative thresholds were not conducted. As the physical activity items were different for the two surveys, observed changes may partly reflect differences in measurement properties. For this reason, and to promote comparability, separate latent class models were estimated for precollege and alumni timepoints using the same modeling structure and conceptual indicators.

Sleep patterns were evaluated differently at each timepoint. The precollege survey included an investigator-developed item and asked respondents to rate their level of agreement with the statement “I usually sleep at least 7–8 h every night”, with responses ranging from strongly disagree to strongly agree. The alumni survey assessed sleep using an item from the American College Health Association’s National College Health Assessment [22], which asked respondents, “how many of the past seven days did you get enough sleep, so that you felt rested when you woke up in the morning?” Sleep quality for precollege and alumni respondents was classified into three ordinal categories: poor (precollege responses: strongly disagree or disagree, alumni responses: 0–2 days), moderate (precollege responses: neutral, alumni responses: 3–5 days), and good (precollege responses: strongly agree or agree, alumni responses: 6–7 days). While these measures of sleep patterns differ, both reflect sleep adequacy.

Body Mass Index (BMI) was calculated using self-reported height and weight (without shoes). Adult BMI cutoffs were used to categorize weight status for both precollege and adult respondents into four categories: underweight, normal weight, overweight, or obese. Although adolescent BMI percentiles are typically used for younger populations, adult BMI cutoffs were selected due to missing age data for 172 individuals, which is required for adolescent classification. A comparison between adolescent percentile-based and adult classifications showed an error rate of 2.9%, with only 23 of 793 individuals classified differently. Specifically, 22 normal-weight adolescents were misclassified as overweight, and one obese adolescent was misclassified as overweight. Because of this low discrepancy, we expect the influence of baseline BMI categorization on longitudinal weight-trajectory assignments and ANCOVA estimates to be minimal. For this reason, we opted to use adult BMI cutoffs in the context of missing age data.

Protocols for both surveys were approved by the Tufts University Institutional Review Board (IRB).

### Data Analyses

Lifestyle behavior patterns at precollege and alumni timepoints were assessed via latent class analysis (LCA) using PROC LCA (Version 1.3.2, 2015, University Park: The Methodology Center, Penn State). Six variables—dietary pattern, fruit intake, vegetable intake, dairy intake, physical activity level, and sleep quality—were used to identify distinct behavioral patterns. Model fit was evaluated using fit statistics (G^2^, AIC, BIC, and adjusted BIC); entropy values indicated moderate separation of classes, supporting hard class assignment for summary (Table 1). A three-class model (healthy, moderately healthy, and minimally healthy) was selected based on its balance and information criteria for each timepoint. Each respondent’s probability of class membership at both timepoints was calculated (Table 2), and respondents were assigned to the class corresponding to the highest probability of a response (Likert style) for a group of questions. The estimated LCA models are presented descriptively (Table 2). To assess longitudinal patterns, combined precollege and adult lifestyle behaviors were categorized as stable healthy, stable moderately healthy, stable minimally healthy, improved, or worsened. 

Weight status patterns were assessed using continuous measures of change in weight (kg) and BMI units as well as categorical patterns—stable healthy weight, stable overweight, stable obesity, improved, or worsened. Among the sixty-eight precollege underweight survey respondents, 13% remained underweight, 81% moved to a healthy weight category, and 6% moved to overweight. Consistent with previous studies [23,24], underweight respondents were excluded from the analysis.

The association between lifestyle trajectories and changes in weight was analyzed using ANCOVA, and pairwise differences in mean weight (kg) and BMI (units) were evaluated using Tukey–Kramer adjustments. As demographic characteristics—age, sex, and race/ethnicity—are known drivers of lifestyle behaviors and weight, analyses examining the association between lifestyle trajectories and changes in weight were adjusted for these sociodemographic variables instead of stratifying by them. We also included graduation year in the model, as a measure of time between the two measurements, along with baseline weight and smoking status. All analyses were conducted using SAS 9.4 (SAS Institute Inc. 2025. SAS/STAT^®^, Cary, NC, USA).

## 3. Results

Respondent characteristics: Between 1996 and 2007, a total of 4783 incoming undergraduates completed the precollege health survey. As seen in Table 3, the longitudinal follow-up sample (*n* = 970) included a slightly lower proportion of males compared to the overall precollege survey respondents. Most demographic characteristics, including race/ethnicity and BMI, were generally comparable between the two groups. The analytic sample was primarily female (60.3%) and non-Hispanic white (77.9%), with a precollege mean age of 17.8 years. Although the longitudinal sample included a greater proportion of non-Hispanic White respondents than the precollege survey respondents (77.9% vs. 70.1%), this difference was not statistically significant. Comparison of baseline characteristics of those who completed the alumni survey with those who did showed significant differences in fruit and dairy intake, suggesting self-selection bias.

**Figure 1:**
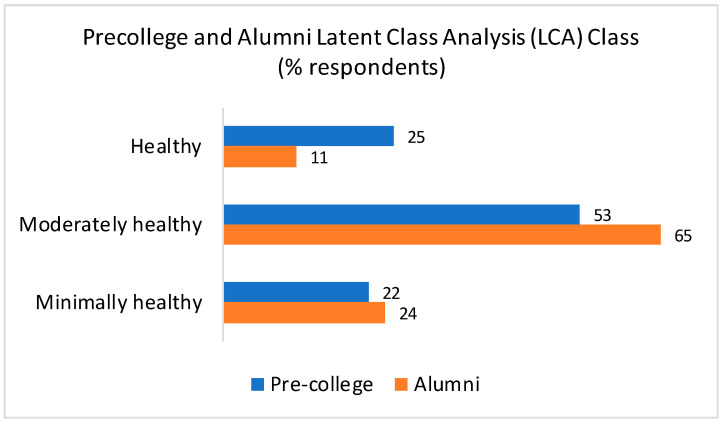

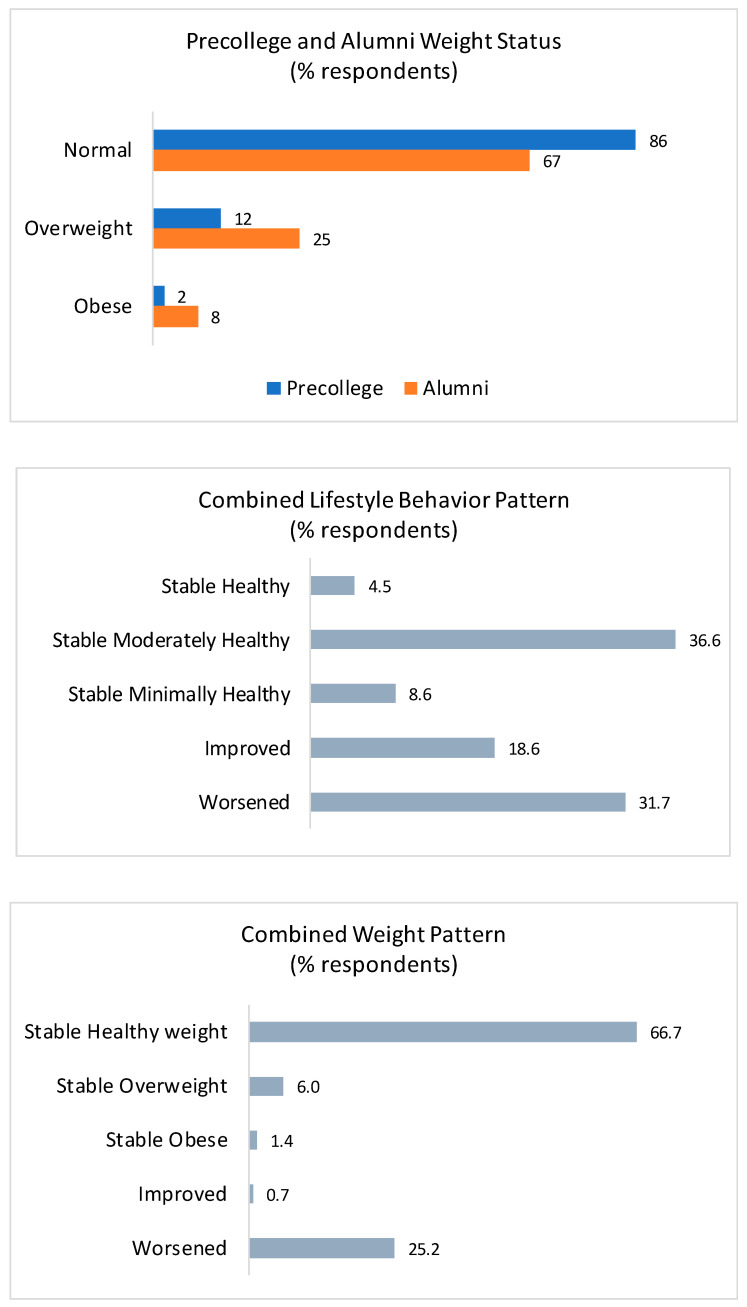
Precollege and adult prevalence of lifestyle behavior classes and weight status and longitudinal trajectories from precollege to adulthood. BMI = Body Mass Index. Lifestyle behavior pattern key: Worsened = healthy to moderately or minimally healthy, moderately healthy to minimally healthy; improved = minimally healthy to moderately healthy or healthy, moderately healthy to healthy. Combined weight pattern key: Worsened = healthy weight to overweight or obese, overweight to obese; improved = obese to overweight or healthy weight, overweight to healthy weight.

Lifestyle behaviors: Substantial shifts in lifestyle behavior patterns were observed between precollege and adulthood. The proportion of respondents classified as having a healthy lifestyle declined from 25% in precollege to 11% in adulthood, while those with a moderately healthy lifestyle increased from 53% to 65%, and those with a minimally healthy lifestyle increased from 22% to 24% (Figure 1). The predominant combined lifestyle behavior pattern was stable moderately healthy (36.7%). While 31.7% of respondents experienced a decline in lifestyle healthfulness (worsened) and moved toward a minimally healthy lifestyle, 18.6% demonstrated improvements and transitioned towards a healthy lifestyle behavior pattern.

Weight status and weight change patterns: The proportion of respondents in the healthy weight category declined from 86% in precollege to 67% in adulthood. Concurrently, the prevalence of overweight more than doubled from 12% to 26%, and the prevalence of obesity increased from 2% in precollege to 8% in adulthood (Figure 1). Distribution of combined weight patterns indicates that 66.7% of respondents remained in a healthy weight category, 25.2% shifted to a higher weight category (gained weight and worsened). Less than one percent (0.7%) transitioned to a lower weight category (improved).

Association between lifestyle behavior change and weight: Baseline sociodemographic characteristics differed minimally between the precollege and longitudinal samples, and we accounted for these factors as covariates in adjusted analyses rather than presenting the sociodemographic characteristics by latent class. Table 4 presents the adjusted associations between lifestyle behavior change and weight trajectories from precollege to adulthood. Among respondents who maintained a stable healthy lifestyle, 79.6% also remained in a stable healthy weight category. In contrast, 56.6% of those with a stable minimally healthy lifestyle maintained a stable healthy weight. Weight gain was particularly pronounced among respondents with a less healthy lifestyle. While 15.9% of respondents with a stable healthy lifestyle transitioned to a higher weight category, 34.9% of those with a stable minimally healthy lifestyle did so. Among those who experienced a decline in lifestyle healthfulness, 24.4% shifted to a higher weight category, and among those who experienced an improvement in lifestyle healthfulness, 23.3% shifted to a higher weight category.

The adjusted average weight gain over the span of 11 to 20 years was 5.7 kg (7.31 kg for men and 4.64 kg for women), with an average BMI increase of 1.86 (2.17 and 1.65 for men and women). Mean weight gain (kg and BMI) was the most pronounced among respondents with a stable minimally healthy lifestyle (8.1 kg [0.54 kg/year], 2.9 BMI units), while those maintaining a stable healthy lifestyle experienced the least amount of weight gain (4.0 kg [0.25 kg/year], 1.4 BMI units).

## 4. Discussion

Emerging adulthood is a critical period for establishing long-term health behaviors, with lifestyle changes during this time having lasting impacts on physical and mental wellbeing [12,25]. Findings from this study indicate that precollege lifestyle behaviors are often maintained into adulthood, and those maintaining healthy lifestyle behaviors or improving lifestyle behaviors were more likely to sustain a healthy weight, while those with persistently poor behaviors experienced greater weight gain. Specifically, two-thirds of all respondents remained in a stable, healthy weight category, 25.2% transitioned to a higher weight category, and less than one percent moved towards a healthy weight category. The average weight gain from precollege to adulthood was 5.7 kg, with the greatest increases among respondents who maintained a stable minimally healthy lifestyle, in contrast to those with a stable healthy lifestyle, who exhibited the lowest gains. While the college environment is likely a driver of behavioral transitions, sociodemographic characteristics and parental influences may also play a critical role in long-term behavioral maintenance [26]. While we did not tabulate the sociodemographic characteristics by latent class, we controlled for these variables in all ANCOVA models to minimize confounding.

This study represents one of the first longitudinal investigations into the associations between lifestyle behavior trajectories and weight patterns from precollege to adulthood. Prior studies have often examined shifts in lifestyle behavior and weight status separately, but few studies have explored these patterns in combination. For example, a systematic review examining weight and lifestyle transition from high school or college to adulthood found that individuals frequently experienced declines in physical activity levels and fruit and dairy consumption, alongside increased weight gain [27]. Our study extends these insights by demonstrating that precollege lifestyle behaviors track into adulthood and are strongly correlated with longer-term (11–20 year) weight outcomes.

Our results are consistent with prior research showing that the transition from adolescence to adulthood is a period of increased weight gain [28]. Data from the Behavioral Risk Factor Surveillance System (1999–2004) illustrated the most substantial increase in obesity rates among 18- to 29-year-olds [29]. Moreover, research from the ADD Health study shows a 5-year incidence of obesity at 13%, with strikingly low remission rates of less than 2% among individuals aged 13 to 20 years [30]. Similarly, the CARDIA study showed that weight gain over five years was more pronounced in men aged 18–24 than in those aged 25–30 [31].

Findings underscore the importance of early, sustained interventions to promote healthy behaviors to mitigate long-term health consequences. In a related study, Laska and colleagues [32] employed LCA to categorize over 2000 college students based on ten lifestyle characteristics (physical activity, fruit and vegetable intake, fast food intake, weight control, smoking, binge drinking, intoxicated sex, drunk driving, poor stress management, and inadequate sleep) and found that only 15.4% of females and 6.2% of males were classified as health conscious, with a higher proportion engaging in high-risk behaviors (24% females and 51% males). Differences between our findings and those reported by Laska et al. [32] may be due to differences in factors like geographic variation, sociodemographic characteristics, timing of assessment, and differences in lifestyle indicators included in the model.

Given that approximately 63% of young adults in the US pursue higher education [10], colleges and universities are uniquely positioned for promoting sustainable, healthy behaviors through environmental, educational, and behavioral strategies. Prior research has highlighted the effectiveness of campus-based interventions and policies designed to support students in adopting healthier behaviors [33,34]. Successful strategies include nutrition interventions such as labels in dining halls and healthy choice marketing campaigns; behavioral nudges such as restricted payment methods for a la carte dining and trayless dining; environmental modifications such as health-themed residence halls and provision of active classroom spaces; and educational programs such as physical activity course requirements, peer mentoring programs, and sleep education courses.

This study is not without limitations. The study participants were drawn from a private university in the Northeast US, which may not reflect the sociodemographic characteristics of precollege students nationwide. In addition, attrition between surveys may have introduced bias: although more than 4000 students completed the precollege survey, only about one-fourth of those individuals participated in the alumni survey. Baseline comparisons of alumni survey respondents and nonrespondents revealed significant differences for some lifestyle behavior variables (e.g., fruit and dairy intake), indicating potential self-selection bias and underestimating adverse trends (as those in the analytic sample had healthier lifestyles than the full cohort at baseline). Several outcome measures were assessed using investigator-developed items, including dietary intake, physical activity at the precollege stage, and sleep at the precollege stage. These indicators may introduce measurement error and limit comparability with more robust and validated measures reported in the literature. Because the physical activity item in the precollege survey did not enable us to differentiate the intensity of reported activities, our physical activity variables were based on total minutes of activity without accounting for potentially important nuances in intensity. Further, due to differences in measures of physical activity and sleep across timepoints, some portion of the observed class transitions may reflect instrument properties, which could bias associations. Harmonization strategies and parallel categorical classifications may also have affected latent class assignment and transitions over time, and results should be interpreted with caution. Similarly, the simplification of dietary patterns (restricted/unrestricted), inability to account for people who do not consume dairy products, and classification of dairy intake (low, medium, high) may not represent the heterogeneity of healthfulness within these categoriesor fully capture the spectrum of dietary preferences, behaviors, and quality. Items were included in a composite measure as contextual indicators and may not serve as reliable standalone indicators of dietary quality. BMI should be interpreted with caution—while it is a widely used measure, it does not distinguish between lean and fat mass. While height and weight measures were self-reported, trained staff also collected these measures for the 1998 cohort, and agreement between these measures was high [35]. Additionally, although the average baseline age of participants was 17.8 years, we used adult BMI cutoffs due to missing age data for 172 individuals. This may introduce minor classification discrepancies; however, comparison showed a low error rate (2.9%), suggesting minimal impact on overall weight status classification. Finally, several unmeasured factors such as stress, social support, and substance use (including drug and alcohol use) were not assessed in the model despite their potential influences on food, physical activity, and sleep behaviors, both independently and as underlying contributors. Omission of these factors may introduce residual confounding.

## 5. Conclusions

These results emphasize the importance of maintaining healthy lifestyle behaviors from precollege through adulthood in supporting favorable weight outcomes. In this study, only a small proportion of respondents maintained a stable healthy lifestyle, and a considerable proportion experienced declines in healthful behaviors that were associated with greater weight gain over time. Conversely, individuals who either sustained or adopted a healthy lifestyle were more likely to maintain a stable weight.

The findings underscore the need for early and continuous interventions to promote healthy habits during the transition from emerging adulthood to adulthood. Given the influential role of higher education institutions in shaping young adults’ habits, implementing evidence-based initiatives such as behavioral nudges, incentives, nutrition labeling, environmental modifications, and education programs in college settings could foster healthy habits and reduce the burden of obesity and related chronic diseases.

Future research should integrate additional factors such as psychosocial stress, social support networks, and environmental determinants that influence weight trajectories. Additionally, inclusion of detailed sociodemographic characteristics can guide tailored program strategies. A deeper understanding of these factors will be critical for developing comprehensive, sustainable strategies aimed at improving long-term health outcomes.

## Figures and Tables

**Table 1:** Relative model fit criteria and classification quality for latent class analysis of lifestyle behaviors of precollege and alumni samples.

Fit Index	Precollege				Alumni			
	Number of Classes							
	2	3	4	5	2	3	4	5
**G^2^**	447.36	414.21	388.08	364.44	471.12	411.74	378.62	354.21
**AIC**	493.36	484.21	482.08	482.44	517.12	481.74	472.62	472.21
**BIC**	605.54	654.91	711.32	770.20	629.30	652.45	701.85	759.97
**BIC (adj)**	532.49	543.75	562.05	582.82	556.25	541.29	552.58	572.58
**Entropy**	0.50	0.60	0.57	0.53	0.51	0.56	0.58	0.52

Fit index values are statistical measurements indicative of model fit. G^2^ is the likelihood ratio chi-square statistic; AIC (Akaike Information Criterion) and BIC (Bayesian Information Criterion) are comparative fit indices, reflecting model complexity and fit, with lower values indicative of better fit. BIC (adj) is an adjusted version of BIC, accounting for sample size. Entropy values (0 to 1) reflect class separation and classification precision.

**Table 2:** Conditional probabilities for lifestyle indicators within each latent lifestyle class at precollege and alumni survey timepoints.

Lifestyle Indicator	Latent Class Analysis Class					
	Precollege			Alumni		
	Healthy	Moderately Healthy	Minimally Healthy	Healthy	Moderately Healthy	Minimally Healthy
	*n* = 246	*n* = 514	*n* = 210	*n* = 106	*n* = 630	*n* = 234
	% Respondents					
**Fruit, servings per day**						
4+ (healthy)	100	0	2.9	72.6	10.5	0
2–3 (moderately healthy)	0	92.0	37.6	21.7	68.4	10.7
0–1 (minimally healthy)	0	8.0	59.5	5.7	21.1	89.3
**Vegetables, servings per day**						
4+ (healthy)	33.3	15.4	0	99.1	29.8	5.1
2–3 (moderately healthy)	53.3	70.6	24.8	0.9	66.0	49.2
0–1 (minimally healthy)	13.4	14.0	75.2	0	4.1	45.7
**Dairy, servings per day**						
4+ (healthy)	39.8	39.1	1.4	30.2	11.4	0.9
2–3 (moderately healthy)	51.6	48.3	62.9	30.2	61.3	21.8
0–1 (minimally healthy)	8.5	12.6	35.7	39.6	27.3	77.3
**Diet type ^1^**						
restricted	26.4	25.7	4.3	48.1	25.6	14.1
unrestricted	73.6	74.3	95.7	51.9	74.4	85.9
**Physical activity, minutes/week ^2^**						
400+ (healthy)	22.0	14.8	6.4	85.9	33.3	15.8
200–400 (moderately healthy)	38.6	39.2	19.1	13.2	42.7	37.6
0–200 (minimally healthy)	39.4	46.0	74.5	0.9	24.0	46.6
**Sleep quality ^3^**						
Poor	59.4	50.0	35.4	14.1	30.6	28.2
Moderate	9.4	14.7	24.9	50.0	53.8	55.6
Good	31.2	35.3	39.7	35.9	15.6	16.2

^1^ Diet type (restricted/not restricted) not provided by two respondents in precollege survey. ^2^ Physical activity data not provided by 30 respondents in precollege survey. ^3^ Sleep quality not provided by 5 respondents in precollege survey. LCA assignments for servings of fruits, vegetables, and dairy per day: healthy = 4+, moderately healthy = 2–3, and minimally healthy = <=1; LCA assignments for diet type: healthy = restricted, moderately healthy = restricted, minimally healthy = unrestricted; LCA assignments for physical activity (minutes per week): healthy = >400, moderately healthy = 200–400, minimally healthy = <200; LCA assignments for precollege sleep quality: I usually sleep at least 8 h every night, poor = strongly disagree/disagree, moderate = neutral, good = strongly agree/agree; LCA assignments for alumni sleep quality: number of days per week slept 8 h, poor = 0 to 2 days, moderate = 3 to 5 days, good = 6 to 7 days.

**Table 3:** Baseline characteristics of precollege respondents and analytic longitudinal samples.

Respondent Characteristic	Precollege Survey Respondents	Analytic Longitudinal Sample
	*n* = 4783	*n* = 970
Sex (%)		
Male	43.8	39.7
Female	56.2	60.3
Race/Ethnicity (%)		
Non-Hispanic White	70.1	77.9
Non-Hispanic Asian	14.0	9.2
Non-Hispanic Black	5.8	2.8
Hispanic	4.7	4.6
Other	5.4	5.5
Age, precollege [mean (SD)] ^1^	17.9 (0.5)	17.8 (0.5)
BMI, precollege [mean (SD)]	22.2 (3.1)	22.4 (2.6)
Male	22.9 (3.2)	22.9 (2.7)
Female	21.7 (2.9)	22.1 (2.6)

^1^ Age not provided by 172 respondents; SD: standard deviation.

**Table 4:** Associations between changes in lifestyle behavior and weight status from precollege to adulthood.

Weight Change from Precollege to Alumni Survey	Lifestyle Behavior Changes from Precollege to Alumni Survey					Overall
	Stable Healthy	Stable Moderately Healthy	Stable Minimally Healthy	Worsened	Improved	
	*n* = 44	*n* = 356	*n* = 83	*n* = 307	*n* = 180	*n* = 970
	% respondents					
**Overall**	4.5	36.7	8.6	31.6	18.6	100.0
**Stable healthy weight**	79.6	65.2	56.6	67.1	70.6	66.7
**Stable overweight**	4.5	6.2	4.8	6.8	5.0	6.0
**Stable obesity**	0.0	2.2	1.2	1.3	0.6	1.4
**Improved weight status**	0.0	0.8	2.4	0.3	0.6	0.7
**Worsened weight status**	15.9	25.6	34.9	24.4	23.3	25.2
	Mean Δ (SE)					
**Adjusted weight (kg) ^1^** **^,^** *	4.0 (1.5)	7.4 (1.0)	8.1 (1.2)	7.1 (1.0)	5.5 (1.0)	6.9 (0.9)
**Adjusted BMI (units) ^1,2^** **^,^** **	1.4 (0.5)	2.5 (0.3)	2.9 (0.4)	2.4 (0.3)	1.8 (0.4)	2.3 (0.3)

Lifestyle behavior key: Worsened = healthy to moderately or minimally healthy, moderately healthy to minimally healthy; improved = minimally healthy to moderately healthy or healthy, moderately healthy to healthy. Weight status key: Worsened = healthy weight to overweight or obese, overweight to obese; improved = obese to overweight or healthy weight, overweight to healthy weight. ^1^ ANCOVA, adjusting for baseline weight (kg), smoking, age (years), sex, race/ethnicity, and year of graduation (measure of time between measurements). The Tukey–Kramer adjustment was used to adjust for multiple comparisons (post hoc adjustment). ^2^ Post hoc adjustment: stable healthy BMI vs. stable minimally healthy; improved BMI vs. stable minimally healthy. * *p* = 0.0245; ** *p* = 0.0265.

## Data Availability

The raw data supporting the conclusions of this article are not publicly available to protect study participants’ privacy; however, they will be made available by the authors upon request.

## Abbreviations

The following abbreviations are used in this manuscript:

LCA	Latent class analysis
BMI	Body Mass Index
